# Cyclic pairwise interaction representing a rock–paper–scissors game maintains the population of the vulnerable yeast *Saccharomyces cerevisiae* within a multispecies sourdough microbiome

**DOI:** 10.1128/spectrum.01370-23

**Published:** 2023-11-02

**Authors:** Mugihito Oshiro, Takeshi Zendo, Yukihiro Tashiro, Jiro Nakayama

**Affiliations:** 1 Central Laboratory of Yamazaki Baking Company Limited, Ichikawa-shi, Chiba, Japan; 2 Laboratory of Soil and Environmental Microbiology, Division of Systems Bioengineering, Department of Bioscience and Biotechnology, Faculty of Agriculture, Graduate School, Kyushu University, Fukuoka, Japan; 3 Laboratory of Microbial Technology, Division of Systems Bioengineering, Department of Bioscience and Biotechnology, Faculty of Agriculture, Graduate School, Kyushu University, Fukuoka, Japan; University of Melbourne, Parkville, Victoria, Australia

**Keywords:** fermented food, mathematical modeling, competition, amensalism, lactic acid bacteria, yeasts

## Abstract

Sourdough starters are spontaneously generated multispecies communities consisting of lactic acid bacteria (LAB) and yeasts. Traditionally used to make baked goods, these communities of microorganisms can be propagated through successive transfers to other containers. Successive transfers result in microbial community dynamics; however, the mechanisms guiding the dynamics are not fully elucidated. This study tracked five species of sourdough LAB-yeast communities (three LAB species and two yeast species) during successive transfers *in vitro* to explore the interspecies interactions driving the multispecies community dynamics. The microbial dynamics were modeled with a generalized Lotka–Volterra (gLV) equation, which can mathematically identify ecological types of interspecies interactions. The gLV model simulated the population dynamics of each species with Pearson’s correlation coefficient values of ≥0.817. Pairwise experiments revealed that competition (negative/negative) predominated among all pairwise interactions (70%), followed by amensalism (negative/neutral) (30%). In the pairwise community, the LAB species *Limosilactobacillus fermentum* suppressed *Saccharomyces cerevisiae* growth through amensalism; however, *S. cerevisiae* population was maintained when the community comprised 3–5 species, including the LAB species *L. fermentum* and *Pediococcus pentosaceus*. The key interspecies interactions for maintaining the *S. cerevisiae* population were theoretically determined using the gLV model; the three identified species interacted non-transitively with *S. cerevisiae* in a cyclic pairwise interaction, metaphorically representing a rock–paper–scissors relationship, which systemically supported the *S. cerevisiae* population in the multispecies community. These theoretical insights indicate that cyclic pairwise interaction is the main driver of LAB-yeast population dynamics, which helps sustain a vulnerable yeast species in a sourdough multispecies community.

**IMPORTANCE:**

Traditionally, multispecies consisting of lactic acid bacteria and yeasts collaboratively engage sourdough fermentation, which determines the quality of the resulting baked goods. Nonetheless, the successive transfer of these microbial communities can result in undesirable community dynamics that prevent the formation of high-quality sourdough bread. Thus, a mechanistic understanding of the community dynamics is fundamental to engineer sourdough complex fermentation. This study describes the population dynamics of five species of lactic acid bacteria-yeast communities *in vitro* using a generalized Lotka–Volterra model that examines interspecies interactions. A vulnerable yeast species was maintained within up to five species community dynamics by obtaining support with a cyclic interspecies interaction. Metaphorically, it involves a rock–paper–scissors game between two lactic acid bacteria species. Application of the generalized Lotka–Volterra model to real food microbiomes including sourdoughs will increase the reliability of the model prediction and help identify key microbial interactions that drive microbiome dynamics.

## OBSERVATION

Traditionally, sourdough starters are used to make a variety of baked goods. They harbor a microbial community consisting of several species of lactic acid bacteria (LAB) and yeasts (1). The multispecies communities of microorganisms in sourdough are generally propagated by successive transfers involving the repeated inoculation of a portion of sourdough into a fresh mixture of flour and water (2): this successive transfer results in microbial community dynamics (3, 4). The mechanisms guiding microbial dynamics are not fully elucidated; however, a possible driver for the community dynamics is an interspecies interaction between sourdough microorganisms (4, 5). Further exploration of the interaction mechanisms underlying the community dynamics will be fundamental to engineer the sourdough microbiome and improve sourdough baking technology.

Numerical studies have characterized sourdough microbial community using culture-dependent and culture-independent methods (6, 7). They captured the fine structure of sourdough microbial communities and presented an inspiration to employ a mathematical approach to gain mechanistic insights into the community dynamics. Some mathematical models are already applied to microbial communities in food fermentations: a system of ordinary differential equations describing two-species dynamics in a yeast community (8), complex growth equations in a two-species community of sourdough (9), and generalized Lotka–Volterra (gLV) equations for multispecies community in cheese (10). Above all, the gLV model is usually applied to time-series data and is a good framework to describe multispecies community dynamics. It can numerically quantify interspecies interactions (11) that cannot be explored by mathematical models previously applied to food fermentation, including the Compertz-based model (12) and logistic model (13). However, to the best of our knowledge, the gLV framework has not been applied to a LAB-yeast community, which is frequently observed during the fermentation of traditional foods (4, 14).

This study applied a gLV model to sourdough multispecies community dynamics. The gLV model was constructed with reference to microbial dynamics in sourdough transferring experiments *in vitro*. The model was verified by comparing the simulation results with various transferring experiment data. Interaction mechanisms of up to five species in a community were numerically inferred according to interspecies interaction values presumed by the model.

An *in vitro* sourdough-modeling medium (15) was used to monitor microbial dynamics driven by all combinations of five strains of five different species of representative sourdough microorganisms (31 transferring experiments in total, namely, 5 experiments in single species, 10 experiments each in pairwise and three species, 5 experiments in four species, and 1 experiment in five species community). All strains were previously isolated from sourdough (16, 17) and consisted of three LAB strains (*Weissella confusa*, *Pediococcus pentosaceus*, and *Limosilactobacillus fermentum*) and two yeast strains (*Saccharomyces cerevisiae* and *Kazachstania unispora*). The five strains showed different population dynamics throughout sourdough transfers (16). Microbial population dynamics were monitored with a CFU counting method (18) using a species-selective plate culture (Table S1). A transferring experiment was initiated by inoculating a small amount of each strain (≤4log CFU/mL) and then transferring it 14–18 times. The parameter values of the constructed gLV model were determined heuristically (Table S2). The intrinsic growth rate in the gLV simulation expressed the maximum CFU-based growth rate (per transfer) intrinsically possessed by each microorganism. Model accuracy was calculated using Pearson’s correlation coefficient and the goodness-of-fit. Strengths of interspecies interactions were presumed based on the interspecies interaction coefficients and the simulated CFUs in the constructed gLV model. Detailed information is available in the Supplemental material.

**Fig 1:**
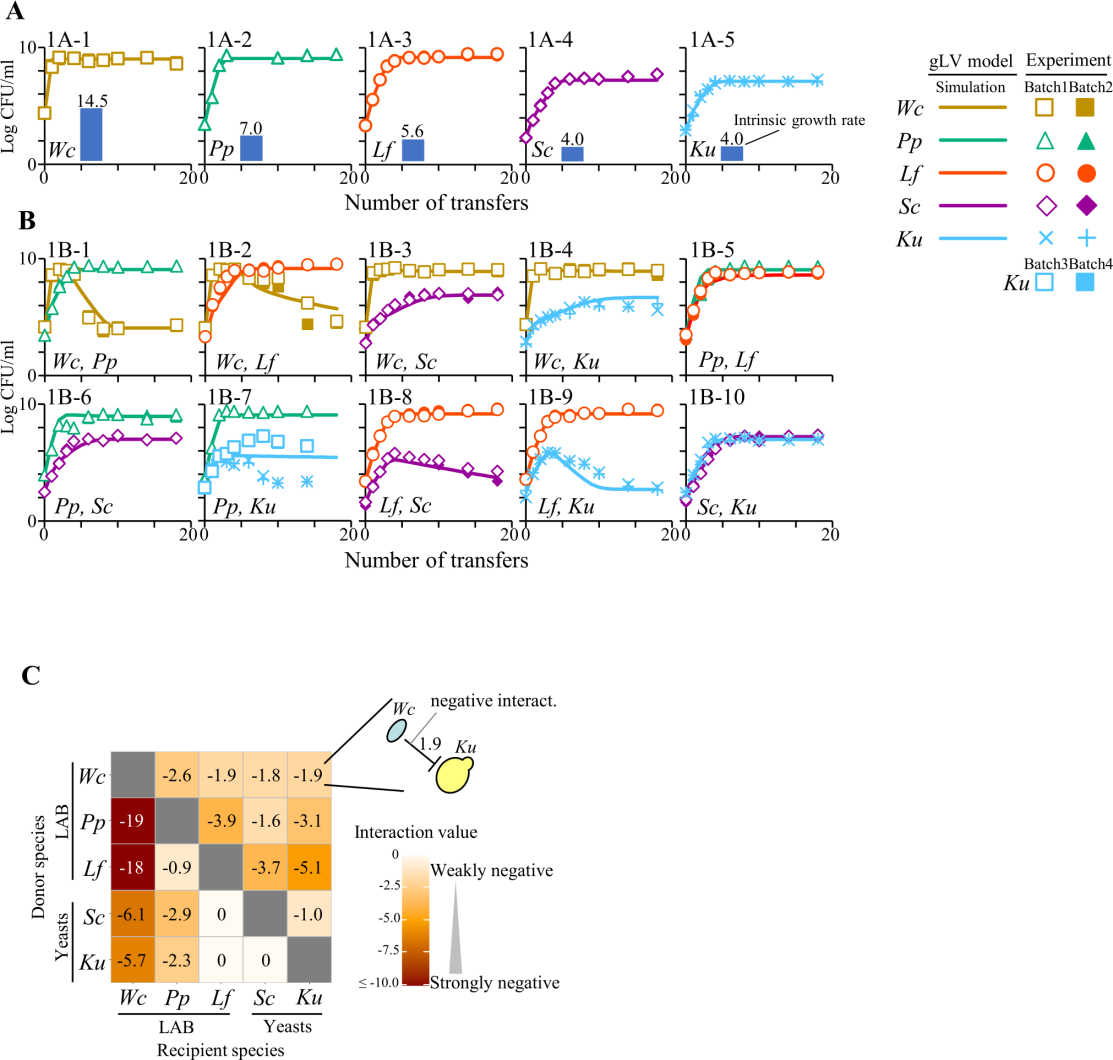
Microbial dynamics and gLV modeling of (A) single and (B) pairwise species assemblages in *in vitro* transferring experiments. Each *in vitro* experiment was independently carried out at least twice (batches 1 and 2) to confirm reproducibility. *Kazachstania unispora* CFU counts in the *Pediococcus pentosaceus* pairwise experiment were performed four times [1B-7 in (B)]. The lines and symbols denote gLV model simulations and experimental data, respectively. The intrinsic growth rate is represented in the blue bar chart inside (A). (C) Matrix of interspecies interaction strengths in all combinations of interspecies interactions. The negative interactions (negative values) are shown in reddish brown tones. A value of 0 represents no interaction. *Wc*, *Weissella confusa*; *Pp*, *Pediococcus pentosaceus*; *Lf*, *Limosilactobacillus fermentum*; *Sc*, *Saccharomyces cerevisiae*; and *Ku*, *Kazachstania unispora*.

In single species transferring experiments, the gLV model captured the population dynamics of each species (Fig. 1A). The intrinsic growth rate of *W. confusa* was 14.5. This value was at least >2 times higher than the rates of other microorganisms, indicating the quick colonizing ability of *W. confusa* in the sourdough environment. The two yeast species grew slower than the three LAB species, with intrinsic growth rates of 4.0 and >5.6, respectively. These *in vitro* growth characteristics are consistent with those of real-world sourdoughs (19).

An analysis of pairwise communities revealed diverse population dynamics in the response of a second microorganism (Fig. 1B). The strong negative interspecies interaction strength of *W. confusa* with *P. pentosaceu*s and *L. fermentum* was simulated as −19 and −18, respectively (Fig. 1C). The gLV model revealed a high prevalence of competition (negative/negative interaction) in 7 out of 10 pairs (70%) (Table 1). The remaining 30% of species pairs exhibit amensalism (negative/neutral). Previous works report LAB-LAB (*Fructilactobacillus sanfranciscensis* and *Lactiplantibacillus plantarum*), LAB-yeast (*F. sanfranciscensi*s and *Kazachstania humilis*), or yeast-yeast (*S. cerevisiae* and *K. humilis*) interactions in sourdough (6, 7). However, knowledge describing which species-pair interacts with each ecological type (competition, amensalism, predation or parasitism, commensalism, and mutualism) is yet to be accumulated.

**TABLE 1:** Ecological types of pairwise interactions

Species pair *^a^*	Ecological interaction type
*W. confusa*/*P*. *pentosaceus*	Competition
*W. confusa*/*L. fermentum*	Competition
*W. confusa*/*S. cerevisiae*	Competition
*W. confusa*/*K. unispora*	Competition
*P. pentosaceus*/*L. fermentum*	Competition
*P. pentosaceus*/*S. cerevisiae*	Competition
*P. pentosaceus*/*K. unispora*	Competition
*L. fermentum*/*S. cerevisiae*	Amensalism
*L. fermentum*/*K. unispora*	Amensalism
*S. cerevisiae*/*K. unispora*	Amensalism

^*a*^ *W*., *Weissella*; *P*., *Pediococcus*; *L*., *Limosilactobacillus*; *S*., *Saccharomyces*; and *K*., *Kazachstania*.

**Fig 2:**
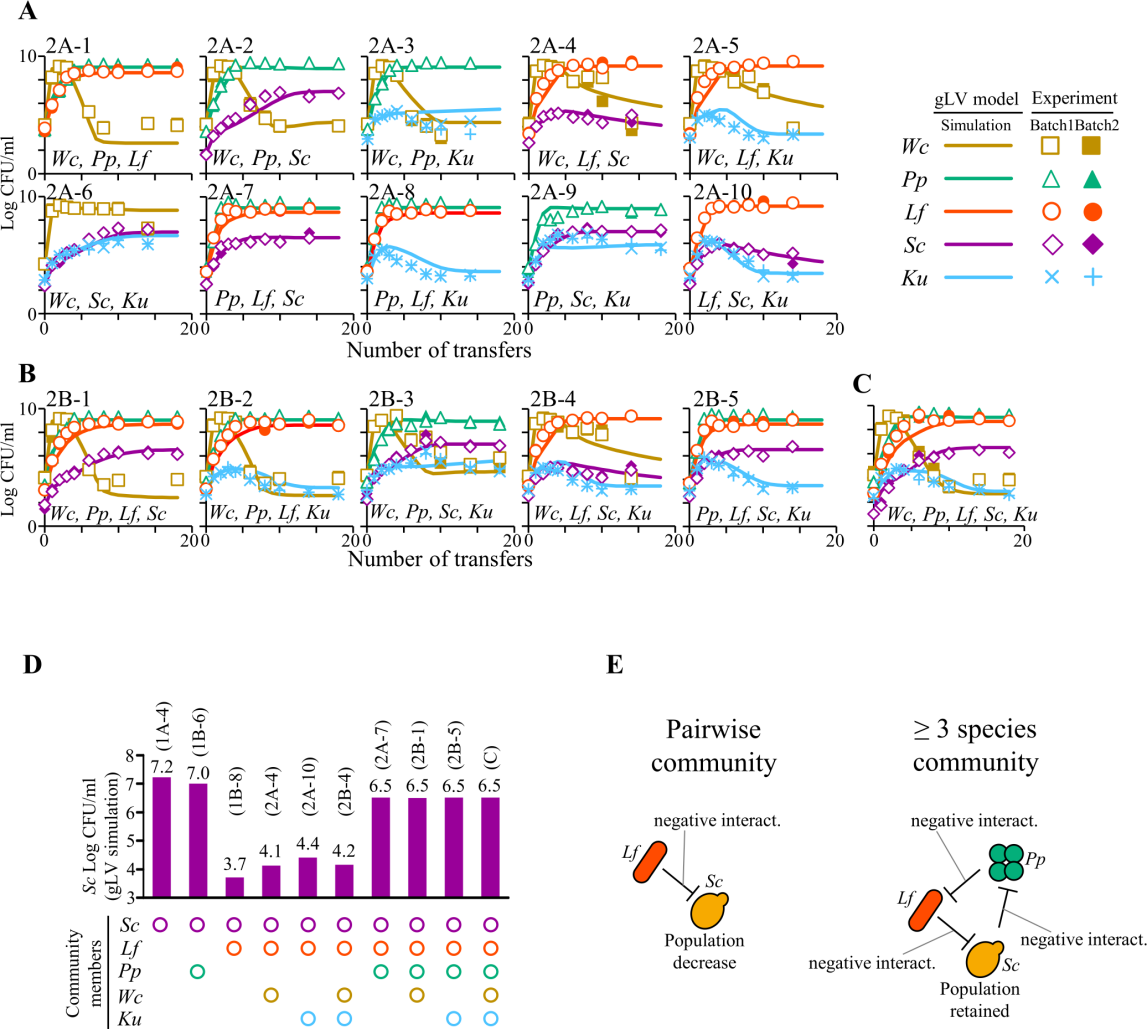
Microbial dynamics and gLV modeling of (A) three, (B) four, and (C) five species assemblages in *in vitro* transferring experiments. Each *in vitro* experiment was independently carried out twice (batches 1 and 2) to confirm reproducibility. The lines and symbols denote gLV model simulations and experimental data, respectively. (D) *Saccharomyces cerevisiae* population after 18 transfers in *Limosilactobacillus fermentum* and *Pediococcus pentosaceus* co-existing communities. The CFU values were simulated by the gLV model. (E) Cyclic pairwise interaction of *S. cerevisiae*, *L. fermentum*, and *P. pentosaceus* in ≥3 species communities. The related interactions in the pairwise community and ≥3 species communities are conceptually illustrated. *Wc*, *Weissella confusa*; *Pp*, *Pediococcus pentosaceus*; *Lf*, *Limosilactobacillus fermentum*; *Sc*, *Saccharomyces cerevisiae*; and *Ku*, *Kazachstania unispora*.

The *S. cerevisiae* population decreased to 3.7log CFU/mL of the gLV model simulation due to the negative effect of *L. fermentum* in the pairwise experiment (Fig. 1B-8), but *S. cerevisiae* retained its population throughout transfers and finally reached 6.5log CFU/mL of the simulation when it was co-cultured with *L. fermentum* and *P. pentosaceus* not only in three species community (Fig. 2A-7) but also up to five species communities (Fig. 2B-1, B-5, C, and D). This mechanism can be explained by non-transitive three species interaction by forming a cyclic pairwise interaction, metaphorically representing a rock–paper–scissors game relationship (20). The three members of the cyclic interaction were *S. cerevisiae*, *L. fermentum*, and *P. pentosaceus* (Fig. S1A). *L. fermentum* defeated *S. cerevisiae*, *S. cerevisiae* defeated *P. pentosaceus*, and *P. pentosaceus* defeated *L. fermentum*, resulting in a cyclic relationship without hierarchy. The gLV model suggested that the cyclic interaction by these members systemically sustained the *S. cerevisiae* population in the multispecies community. Real sourdough microbiome might comprise those three members simultaneously when *S. cerevisiae* counts reach ≥6.8log CFU/g and maintain its population there (19). Similar cyclic trio interactions have maintained a bacterial community (21). Meanwhile, the other nine communities of three species combinations were transitive interactions (Fig. S1B).

Even in the complex model, all Pearson’s correlation coefficient values were ≥0.817 (median = 0.983), whereas all goodness-of-fits were ≤0.973 (median = 0.140) (Fig. S2; Tables S3 and S4), supporting the accuracy of the gLV model. Serially transferring experiments were reasonably described by the gLV equation (22).

A gLV model is informative of key microbial interactions underlying the community dynamics; however, the model does not consider additional factors such as environmental pH changes (17), or maltose assimilating abilities of microorganisms (19): both factors are known to influence the sourdough community. Another limitation is that this study applied the gLV model to only one data set of five microbial species assembly in *in vitro* sourdough. Further applications of the gLV model to real microbial communities of LAB-yeast fermented foods including sourdoughs increase the reliability of model prediction and help identify key microbial interactions that drive food microbiome dynamics.

In summary, the constructed gLV model well simulated a total of 31 experiments of diverse dynamics of LAB-yeast communities observed in sourdough transferring experiments *in vitro*. The mathematical model revealed that the cyclic pairwise interaction formed by three species consisting of *S. cerevisiae*, *L. fermentum*, and *P. pentosaceus* drove the multispecies community dynamics, and the cyclic interaction maintained the vulnerable *S. cerevisiae* population within up to five species community dynamics.

## SUPPLEMENTAL MATERIAL

Supplemental figures spectrum.01370-23-s0001.pdf Fig. S1 and S2.

Supplemental material spectrum.01370-23-s0002.doc Tables S1 to S4; detailed Materials and Methods.

ASM does not own the copyrights to Supplemental Material that may be linked to, or accessed through, an article. The authors have granted ASM a non-exclusive, world-wide license to publish the Supplemental Material files. Please contact the corresponding author directly for reuse.
