# Supplementary material for: Cyclic pairwise interaction representing a rock–paper–scissors game maintains the population of the vulnerable yeast Saccharomyces cerevisiae within a multispecies sourdough microbiome
Source: Microbiol Spectr. 2023 Nov 2;11(6):e01370-23. doi: 10.1128/spectrum.01370-23 (PMC10714952; doi:10.1128/spectrum.01370-23)
Supplement: Supplemental figures — Fig. S1 and S2. [file spectrum.01370-23-s0001.pdf]

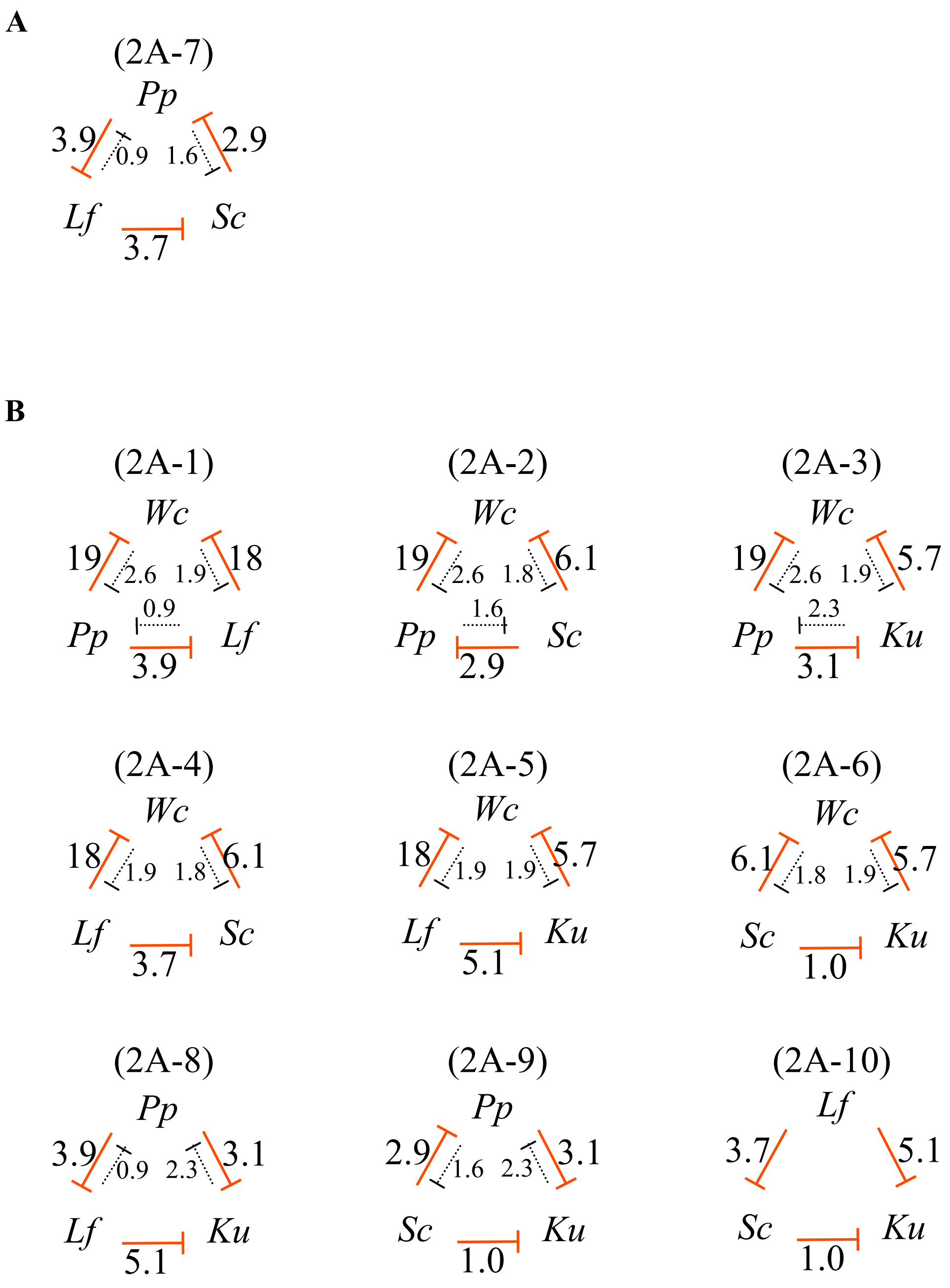

FIG S1 Three species interaction networks in *in vitro* experiments. (A) Non-transitive (cyclic) interactions. (B) Ttransitive interactions. Each value of interspecies interaction strengths is shown with the direction. The all values mean negative interactions. The larger value means the stonger negative interaction. Between two species, the direction of the larger negative-interaction is colored with red. The letters in parentheses show the corresponded figure in Fig. 2A. *Wc*, *Weissella confusa*; *Pp*, *Pediococcus pentosaceus*; *Lf*, *Limosilactobacillus fermentum*; *Sc*, *Saccharomyces cerevisiae*; *Ku*, *Kazachstania unispora*.

Figure S1 Oshiro et al.

C

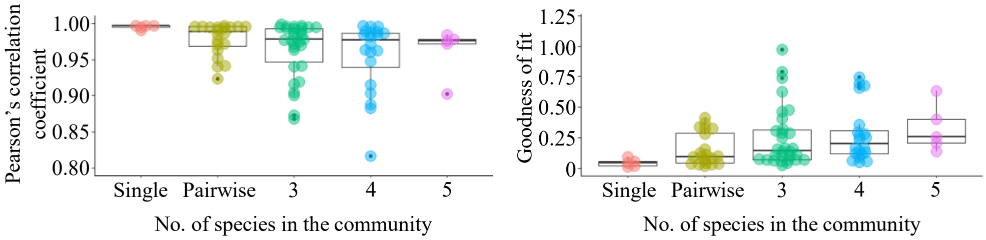

FIG S2 Box plots of Pearson's correlation coefficient (left) and goodness of fit (right) for each *in vitro* transferring experiment. These values were evaluated and plotted by each species of each community

Figure S2 Oshiro et al.
