# Supplementary material for: Cyclic pairwise interaction representing a rock–paper–scissors game maintains the population of the vulnerable yeast Saccharomyces cerevisiae within a multispecies sourdough microbiome
Source: Microbiol Spectr. 2023 Nov 2;11(6):e01370-23. doi: 10.1128/spectrum.01370-23 (PMC10714952; doi:10.1128/spectrum.01370-23)
Supplement: Supplemental material — Tables S1 to S4; detailed Materials and Methods. [file spectrum.01370-23-s0002.doc]

Supplemental material for

**Cyclic pairwise interaction representing a rock–paper–scissors game maintains the population of the vulnerable yeast *Saccharomyces cerevisiae* within a multispecies sourdough microbiome**

TABLE S1. List of species-selective agar plates used.

| Species | Medium | Cultivation temperature (°C) |
| --- | --- | --- |
| *Weissella confusa* | BL agar (Eiken Chemical)  with 10 g/L sucrose and 0.1 g/L cycloheximide, pH 9 | 25 |
| *Pediococcus pentosaceus* | MRS agar (Merck) with 60 g/L NaCl and 0.1 g/L cycloheximide | 37 |
| *Limosilactobacillus fermentum* | MRS agar (Merck) with 0.1 g/L cycloheximide | 45 |
| *Saccharomyces cerevisiae* | Potato dextrose agar (Eiken Chemical) with 0.1 g/L chloramphenicol | 37 |
| *Kazachstania unispora* | Potato dextrose agar (Eiken Chemical) with 0.1 g/L chloramphenicol  and 0.1 g/L cycloheximide | 25 |

TABLE S2. Summary of the parameter values obtained from generalized Lotka–Volterra modeling *^a^*.

| Parameter *^a^* | Value |
| --- | --- |
| 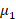 | 14.5 |
| 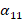 | −1.5×10^−8^ |
| 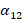 | −1.6×10^−8^ |
| 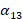 | −1.2×10^−8^ |
| 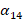 | −3.63×10^−7^ |
| 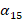 | −4.35×10^−7^ |
| 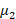 | 7.0 |
| 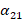 | −3.0×10^−9^ |
| 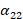 | −6.0×10^−9^ |
| 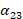 | −1.0×10^−9^ |
| 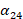 | −1.75×10^−7^ |
| 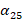 | −1.75×10^−7^ |
| 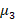 | 5.55 |
| 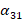 | −2.0×10^−9^ |
| 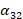 | −3.0×10^−9^ |
| 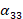 | −4.0×10^−9^ |
| 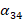 | 0 |
| 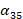 | 0 |
| 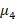 | 4.0 |
| 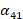 | −2.0×10^−9^ |
| 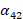 | −1.0×10^−9^ |
| 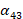 | −2.0×10^−9^ |
| 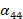 | −2.22×10^−7^ |
| 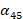 | 0 |
| 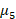 | 4.0 |
| 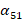 | −2.0×10^−9^ |
| 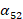 | −3.0×10^−9^ |
| 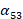 | −3.0×10^−9^ |
| 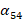 | −5.7×10^−8^ |
| 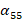 | −2.86×10^−7^ |
| 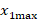 | 1.0×10^9^ |
| 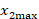 | 1.2×10^9^ |
| 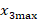 | 1.5×10^9^ |
| 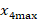 | 1.7×10^7^ |
| 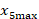 | 1.3×10^7^ |

*^a^* 1
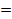
*Weissella confusa*, 2
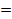
*Pediococcus pentosaceus*, 3
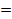
*Limosilactobacillus fermentum*, 4
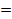
*Saccharomyces cerevisiae*, 4
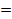
*Kazachstania unispora*.

TABLE S3. Pearson’s correlation coefficients between the gLV model simulation results and *in vitro* transferring experimental data *^a^*.

| Single | | Pairwise | | | 3 species | | | 4 species | | | 5 species | | |
| --- | --- | --- | --- | --- | --- | --- | --- | --- | --- | --- | --- | --- | --- |
| Sp. | Value | Sp. | Value | Community | Sp. | Value | Community | Sp. | Value | Community | Sp. | Value | Community |
| *Wc* | 0.990 | *Wc* | 0.972 | *Wc, Pp* | *Wc* | 0.976 | *Wc, Pp, Lf* | *Wc* | 0.962 | *Wc, Pp, Lf, Sc* | *Wc* | 0.984 | *Wc, Pp, Lf, Sc, Ku* |
| *Pp* | 0.994 | *Pp* | 0.996 |  | *Pp* | 0.983 |  | *Pp* | 0.996 |  | *Pp* | 0.978 |  |
| *Lf* | 0.997 | *Wc* | 0.941 | *Wc, Lf* | *Lf* | 0.995 |  | *Lf* | 0.988 |  | *Lf* | 0.976 |  |
| *Sc* | 0.997 | *Lf* | 0.976 |  | *Wc* | 0.994 | *Wc, Pp, Sc* | *Sc* | 0.986 |  | *Sc* | 0.972 |  |
| *Ku* | 0.997 | *Wc* | 0.996 | *Wc, Sc* | *Pp* | 0.997 |  | *Wc* | 0.963 | *Wc, Pp, Lf, Ku* | *Ku* | 0.903 |  |
|  |  | *Sc* | 0.986 |  | *Sc* | 0.967 |  | *Pp* | 0.996 |  |  |  |  |
|  |  | *Wc* | 0.991 | *Wc, Ku* | *Wc* | 0.963 | *Wc, Pp, Ku* | *Lf* | 0.984 |  |  |  |  |
|  |  | *Ku* | 0.970 |  | *Pp* | 0.999 |  | *Ku* | 0.915 |  |  |  |  |
|  |  | *Pp* | 0.952 | *Pp, Lf* | *Ku* | 0.900 |  | *Wc* | 0.947 | *Wc, Pp, Sc, Ku* |  |  |  |
|  |  | *Lf* | 0.995 |  | *Wc* | 0.916 | *Wc, Lf, Sc* | *Pp* | 0.986 |  |  |  |  |
|  |  | *Pp* | 0.963 | *Pp, Sc* | *Lf* | 0.978 |  | *Sc* | 0.982 |  |  |  |  |
|  |  | *Sc* | 0.984 |  | *Sc* | 0.940 |  | *Ku* | 0.817 |  |  |  |  |
|  |  | *Pp* | 0.996 | *Pp, Ku* | *Wc* | 0.905 | *Wc, Lf, Ku* | *Wc* | 0.888 | *Wc, Lf, Sc, Ku* |  |  |  |
|  |  | *Ku* | 0.940 |  | *Lf* | 0.969 |  | *Lf* | 0.972 |  |  |  |  |
|  |  | *Lf* | 0.995 | *Lf, Sc* | *Ku* | 0.873 |  | *Sc* | 0.883 |  |  |  |  |
|  |  | *Sc* | 0.996 |  | *Wc* | 0.941 | *Wc, Sc, Ku* | *Ku* | 0.904 |  |  |  |  |
|  |  | *Lf* | 0.995 | *Lf, Ku* | *Sc* | 0.996 |  | *Pp* | 0.997 | *Pp, Lf, Sc, Ku* |  |  |  |
|  |  | *Ku* | 0.923 |  | *Ku* | 0.977 |  | *Lf* | 0.989 |  |  |  |  |
|  |  | *Sc* | 0.997 | *Sc, Ku* | *Pp* | 0.995 | *Pp, Lf, Sc* | *Sc* | 0.984 |  |  |  |  |
|  |  | *Ku* | 0.993 |  | *Lf* | 0.984 |  | *Ku* | 0.960 |  |  |  |  |
|  |  |  |  |  | *Sc* | 0.987 |  |  |  |  |  |  |  |
|  |  |  |  |  | *Pp* | 0.996 | *Pp, Lf, Ku* |  |  |  |  |  |  |
|  |  |  |  |  | *Lf* | 0.989 |  |  |  |  |  |  |  |
|  |  |  |  |  | *Ku* | 0.868 |  |  |  |  |  |  |  |
|  |  |  |  |  | *Pp* | 0.972 | *Pp, Sc, Ku* |  |  |  |  |  |  |
|  |  |  |  |  | *Sc* | 0.992 |  |  |  |  |  |  |  |
|  |  |  |  |  | *Ku* | 0.919 |  |  |  |  |  |  |  |
|  |  |  |  |  | *Lf* | 0.993 | *Lf, Sc, Ku* |  |  |  |  |  |  |
|  |  |  |  |  | *Sc* | 0.992 |  |  |  |  |  |  |  |
|  |  |  |  |  | *Ku* | 0.975 |  |  |  |  |  |  | Overall |
| Min | 0.990 |  | 0.923 |  |  | 0.868 |  |  | 0.817 |  |  | 0.903 | 0.817 |
| Max | 0.997 |  | 0.997 |  |  | 0.999 |  |  | 0.997 |  |  | 0.984 | 0.999 |
| Median | 0.997 |  | 0.989 |  |  | 0.978 |  |  | 0.977 |  |  | 0.976 | 0.983 |
| Average | 0.995 |  | 0.978 |  |  | 0.964 |  |  | 0.955 |  |  | 0.963 | 0.967 |

*^a^* The value was calculated for each species of each community. Sp., species; *Wc*, *Weissella confusa*; *Pp*, *Pediococcus pentosaceus*; *Lf*, *Limosilactobacillus fermentum*; *Sc*, *Saccharomyces cerevisiae*; *Ku*, *Kazachstania unispora*.

TABLE S4. Goodness of fits between the gLV model simulation results and *in vitro* transferring experimental data *^a^*.

| Single | | Pairwise | | | 3 species | | | 4 species | | | 5 species | | |
| --- | --- | --- | --- | --- | --- | --- | --- | --- | --- | --- | --- | --- | --- |
| Sp. | Value | Sp. | Value | Community | Sp. | Value | Community | Sp. | Value | Community | Sp. | Value | Community |
| *Wc* | 0.047 | *Wc* | 0.325 | *Wc, Pp* | *Wc* | 0.973 | *Wc, Pp, Lf* | *Wc* | 0.699 | *Wc, Pp, Lf, Sc* | *Wc* | 0.403 | *Wc, Pp, Lf, Sc, Ku* |
| *Pp* | 0.092 | *Pp* | 0.072 |  | *Pp* | 0.110 |  | *Pp* | 0.060 |  | *Pp* | 0.209 |  |
| *Lf* | 0.021 | *Wc* | 0.417 | *Wc, Lf* | *Lf* | 0.071 |  | *Lf* | 0.249 |  | *Lf* | 0.635 |  |
| *Sc* | 0.055 | *Lf* | 0.340 |  | *Wc* | 0.067 | *Wc, Pp, Sc* | *Sc* | 0.088 |  | *Sc* | 0.259 |  |
| *Ku* | 0.012 | *Wc* | 0.019 | *Wc, Sc* | *Pp* | 0.138 |  | *Wc* | 0.676 | *Wc, Pp, Lf, Ku* | *Ku* | 0.141 |  |
|  |  | *Sc* | 0.112 |  | *Sc* | 0.304 |  | *Pp* | 0.048 |  |  |  |  |
|  |  | *Wc* | 0.040 | *Wc, Ku* | *Wc* | 0.789 | *Wc, Pp, Ku* | *Lf* | 0.238 |  |  |  |  |
|  |  | *Ku* | 0.158 |  | *Pp* | 0.057 |  | *Ku* | 0.264 |  |  |  |  |
|  |  | *Pp* | 0.324 | *Pp, Lf* | *Ku* | 0.156 |  | *Wc* | 0.658 | *Wc, Pp, Sc, Ku* |  |  |  |
|  |  | *Lf* | 0.081 |  | *Wc* | 0.630 | *Wc, Lf, Sc* | *Pp* | 0.125 |  |  |  |  |
|  |  | *Pp* | 0.280 | *Pp, Sc* | *Lf* | 0.164 |  | *Sc* | 0.147 |  |  |  |  |
|  |  | *Sc* | 0.097 |  | *Sc* | 0.071 |  | *Ku* | 0.282 |  |  |  |  |
|  |  | *Pp* | 0.126 | *Pp, Ku* | *Wc* | 0.738 | *Wc, Lf, Ku* | *Wc* | 0.749 | *Wc, Lf, Sc, Ku* |  |  |  |
|  |  | *Ku* | 0.097 |  | *Lf* | 0.464 |  | *Lf* | 0.358 |  |  |  |  |
|  |  | *Lf* | 0.039 | *Lf, Sc* | *Ku* | 0.317 |  | *Sc* | 0.164 |  |  |  |  |
|  |  | *Sc* | 0.093 |  | *Wc* | 0.291 | *Wc, Sc, Ku* | *Ku* | 0.293 |  |  |  |  |
|  |  | *Lf* | 0.033 | *Lf, Ku* | *Sc* | 0.136 |  | *Pp* | 0.132 | *Pp, Lf, Sc, Ku* |  |  |  |
|  |  | *Ku* | 0.370 |  | *Ku* | 0.098 |  | *Lf* | 0.167 |  |  |  |  |
|  |  | *Sc* | 0.029 | *Sc, Ku* | *Pp* | 0.112 | *Pp, Lf, Sc* | *Sc* | 0.055 |  |  |  |  |
|  |  | *Ku* | 0.044 |  | *Lf* | 0.289 |  | *Ku* | 0.111 |  |  |  |  |
|  |  |  |  |  | *Sc* | 0.042 |  |  |  |  |  |  |  |
|  |  |  |  |  | *Pp* | 0.100 | *Pp, Lf, Ku* |  |  |  |  |  |  |
|  |  |  |  |  | *Lf* | 0.163 |  |  |  |  |  |  |  |
|  |  |  |  |  | *Ku* | 0.475 |  |  |  |  |  |  |  |
|  |  |  |  |  | *Pp* | 0.252 | *Pp, Sc, Ku* |  |  |  |  |  |  |
|  |  |  |  |  | *Sc* | 0.062 |  |  |  |  |  |  |  |
|  |  |  |  |  | *Ku* | 0.410 |  |  |  |  |  |  |  |
|  |  |  |  |  | *Lf* | 0.075 | *Lf, Sc, Ku* |  |  |  |  |  |  |
|  |  |  |  |  | *Sc* | 0.025 |  |  |  |  |  |  |  |
|  |  |  |  |  | *Ku* | 0.070 |  |  |  |  |  |  | Overall |
| Min | 0.012 |  | 0.019 |  |  | 0.025 |  |  | 0.048 |  |  | 0.141 | 0.012 |
| Max | 0.092 |  | 0.417 |  |  | 0.973 |  |  | 0.749 |  |  | 0.635 | 0.973 |
| Median | 0.047 |  | 0.097 |  |  | 0.147 |  |  | 0.203 |  |  | 0.259 | 0.140 |
| Average | 0.045 |  | 0.155 |  |  | 0.255 |  |  | 0.278 |  |  | 0.329 | 0.227 |

*^a^* The value is calculated for each species of each community. Sp., species; *Wc*, *Weissella confusa*; *Pp*, *Pediococcus pentosaceus*; *Lf*, *Limosilactobacillus fermentum*; *Sc*, *Saccharomyces cerevisiae*; *Ku*, *Kazachstania unispora*.

**Detailed Materials and Methods**

***In vitro* sourdough transferring experiments.**

**(i) Strains and medium.**

Five strains of five different species (*Weissella confusa*, *Pediococcus pentosaceus*, *Limosilactobacillus fermentum*, *Saccharomyces cerevisiae*, and *Kazachstania unispora*) were previously isolated from sourdoughs (1, 2). These were genetically identified using rRNA gene sequencing and housekeeping gene sequencing, if necessary. An *in vitro* sourdough medium, wheat sourdough simulation medium (WSSM) previously reported by Vrancken et al. (3), was prepared for all *in vitro* experiments.

**(ii) Transferring conditions.**

The transferring experiment was initiated by inoculating single species of two to five combined species with ≤4 log CFU of each species into 1 mL WSSM. Fermentation was carried out at 30°C for 8 h; the fermented product was then stored at 4°C until the next transfer. Each transfer involved inoculation of 20% v/v of the previous sourdough starter into fresh WSSM medium, and this was repeated 14 to 18 times. Each transfer was also carried out separately with the initial species, at a concentration of ≤4 log CFU/mL of each strain, in order to simulate microbial contamination from non-sterile ingredients and environment in actual sourdough. The CFUs of each species were periodically counted during transfers using a selective agar plate (Table S1). The drop-plating method was used for high-throughput CFU counting (4). The CFUs were counted two times per sample, and the average values were stated for each sample. Each transferring experiment was performed at least twice to confirm the reproducibility.

**Generalized Lotka-Volterra modeling**

The generalized Lotka-Volterra (gLV) model was employed to describe the time-course of CFU counts in all *in vitro* experiments. The gLV model is a set of coupled ordinary differential equations that describe the temporal dynamics in species abundance (5). The model equation is as follows:


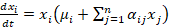
,

where
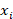
,
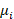
,
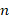
,
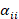
, and
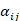
 represent CFU counts of species
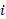
, intrinsic growth rate of species
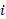
, the number of species in the experiment, intraspecies interaction coefficients of species
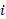
, and interspecies interaction coefficients from species
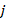
 to
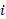
, respectively. An
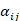
 < 0 denotes a negative interspecies interaction. An
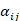
 = 0 means no interspecies interaction. The values were inferred (Table S3) following a preliminary inspection of the *in vitro* experimental data shown in Fig. 1.

**Validation of the gLV model**

The CFU count data of *in vitro* experiments and model simulations were normalized to logarithmic values before the validation. Pearson’s correlation coefficient (6) and the goodness-of-fit (gf) (6) were calculated by comparing the model simulation results with transferring experimental data at each time point of the transfers. The gf of the model was calculated using the following equation:


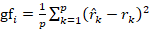
,

where
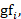

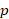
,
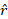
, and
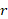
 denote the gf of species
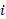
, time point, model simulated result, and experimental value, respectively. Box plots were created with the R packages tidyverse and ggbeeswarm.

**Presumption of interspecies interaction strengths**

The interspecies interaction strength (IIS) was calculated based on the parameter values in the gLV model using the following equation:


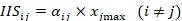
,

where,
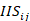
,
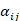
, and
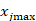
 denote IIS directed from species
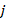
 to
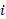
, interspecies interaction coefficients from species
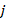
 to
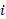
, and the simulated CFU counts of species
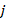
 at 18 transfer times in single species
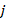
 transferring-simulation, respectively. An
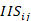
 < 0 represents a negative interspecies interaction. An
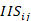
 = 0 means no interaction. The Heatmap was created with the R packages ggplot2 and dplyr.

**REFERENCES**

1. Oshiro M, Momoda R, Tanaka M, Zendo T, Nakayama J. 2019. Dense tracking of the dynamics of the microbial community and chemicals constituents in spontaneous wheat sourdough during two months of backslopping. J Biosci Bioeng 128:170–176.

2. Oshiro M, Tanaka M, Zendo T, Nakayama J. 2020. Impact of pH on succession of sourdough lactic acid bacteria communities and their fermentation properties. Biosci Microbiota Food Health 39:152–159.

3. Vrancken G, Rimaux T, De Vuyst L, Leroy F. 2008. Kinetic analysis of growth and sugar consumption by *Lactobacillus fermentum* IMDO 130101 reveals adaptation to the acidic sourdough ecosystem. Int J Food Microbiol 128:58–66.

4. Herigstad B, Hamilton M, Heersink J. 2001. How to optimize the drop plate method for enumerating bacteria. J Microbiol Methods 44:121–129.

5. Gonze D, Coyte KZ, Lahti L, Faust K. 2018. Microbial communities as dynamical systems. Curr Opin Microbiol 44:41–49.

6. Venturelli OS, Carr AC, Fisher G, Hsu RH, Lau R, Bowen BP, Hromada S, Northen T, Arkin AP. 2018. Deciphering microbial interactions in synthetic human gut microbiome communities. Mol Syst Biol 14:e8157.
